# The relationship between body image and emotional and cognitive impairment after brain damage: A preliminary study

**DOI:** 10.1002/brb3.2181

**Published:** 2021-05-18

**Authors:** Francesco Corallo, Dominique Tarda, Valentina Coppola, Lilla Bonanno, Viviana Lo Buono, Rosanna Palmeri, Maria Cristina De Cola, Marcella Di Cara, Laura Romeo, Loredana Raciti, Antonino Todaro, Anna Lisa Logiudice, Placido Bramanti, Silvia Marino, Caterina Formica

**Affiliations:** ^1^ IRCCS Centro Neurolesi “Bonino‐Pulejo” Italy

**Keywords:** anxiety, body image, depression, mood, self‐perception

## Abstract

**Introduction:**

The image of one's own body derives from experimentation of one's own body pattern. The emotional experience can lead to a real or distorted self‐representation. After brain damage, a disorder of body image is frequent. The purpose of this study was to investigate the role of body image following acquired brain injury (ABI).

**Methods:**

Forty‐six hospitalized patients were enrolled and subdivided into two groups depending on the etiology of the damage: traumatic or vascular. For each group, we considered their cognitive level and mood. Patients underwent a broad battery of tests to investigate different domains: Montreal Cognitive Assessment (MoCA); Beck's Depression Inventory (BDI‐II); Hamilton Rating Scale for Anxiety (HAM‐A); Clinical Insight Rating Scale (CIRS); Body Image Scale (BIS); Human Figure Drawing (HFD). The latter was used to assess the implicit body image of each patient.

**Results:**

Both groups showed a significant relationship between BDI‐II and BIS. A positive correlation was found between BIS and HAM‐A, but only in the traumatic group. We showed a positive correlation between MoCA and HFD. In addition, we observed some subitems of MoCA as predictive variables in HFD, which differ in the two groups. In a traumatic group, the visuospatial domain is predictive in HFD, as well as age of patients’ and education. In the vascular groups, orientation, naming, abstraction, and language domains are instead predictive.

**Conclusions:**

The results confirm the crucial role of the cognitive level and mood on self‐perception.

## Graphical abstract

Body image is a person's perception of the aesthetics or sexual attractiveness of their own body. After brain damage, the person is subjected to several physical changes with heavy consequences on both physical and emotional domains. It can lead to a dysfunction of the body image and to the risk to compromise cognitive recovery. Notably, this study confirms the relationship between body image and cognitive deficits in ABI patients, especially noticeable in vascular patients.

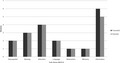

## 1 INTRODUCTION

Acquired brain injury (ABI) is a generic term used to indicate several clinical conditions, including traumatic brain injury (TBI) and stroke. ABI causes a variety of neurological symptoms manifested by alteration of cognition, affectivity, and sensory‐motor capacity (Bartuccio et al., 2012). Frequently, patients with ABI show deficits in the domains of attention and memory. In particular, difficulties in concentration, distractibility, and forgetfulness are disabling factors (Hinkeldey & Corrigan, 1990; Mateer et al., 1987). Usually, these difficulties are associated with executive dysfunction (Mateer & Map ou, 1996).

The physical and psychological consequences of ABI represent a real challenge for the individual (Hackett et al., 2005). The hospitalization and the slow process of recovery, associated with the awareness of cognitive and physical impairment, may be cause emotional disorders (Gainotti, 1993). In fact, very often, individuals with ABI have high levels of anxiety and depression, with a prevalence of 17%–29% for anxiety (Broomfield et al., 2015) and a prevalence of 23%–50% for depression (Lenzi et al., 2008; Maaijwee et al., 2016).

In consideration of the numerous physical changes after ABI, patients are called to accept their new body and to create a new body image. The somatosensory neurological process, physical and emotional disorders cause a dysfunction of the body image (Corbett & Shah, 1996). In a longitudinal study, Kinsella and Ford (1980) demonstrated that body image disorders, accompanied by hemiparesis, visuospatial, and attention disorders, become an important cognitive barrier for a successful recovery.

Many ABI patients have insufficient insight and emotional distress. However, the study conducted by Heilbronner et al. (1989) showed a significant correlation between disability perceived, presence of depressive symptoms, and the effect of rehabilitative treatment. Patients with greater self‐awareness showed high levels of depression.

This study aims to explore the relationship between body image and emotional and cognitive impairment in ABI patients.

## 2 MATERIALS AND METHODS

The study was designed as retrospective cross‐sectional on a sample of ABI patients hospitalized at the Rehabilitation Unit for Severe Acquired Brain Injuries of the IRCCS Centro Neurolesi “Bonino‐Pulejo” of Messina.

Forty‐six patients (26 men, 20 women) meanly aged 55.33 ± 17.97 years were found eligible for this study according to the inclusion/exclusion criteria. Notably, inclusion criteria were: ABI diagnosis, age over 17 years, and absence of any neurological or psychiatric deficit, whereas exclusion criteria were: presence of premorbid psychological symptoms, more than 3 months from the brain injury occurrence, presence of prosopagnosia or neglect, presence of paresis.

This retrospective cohort study did not require the approval of the Ethics Committee, in accordance with the current rules of our hospital. However, all participants were contacted and provided written informed consent to enter the study.

Participants were divided into two groups according to etiology, that is, traumatic group and vascular group. Both groups were submitted the same neuropsychological assessment including several tests. The Montreal Cognitive Assessment (MoCA) was used to assess different cognitive domains: orientation, visuospatial abilities, naming, language, memory, abstraction, and attention skills. A score <26 indicates a cognitive deficit (Nasreddine et al., 2005). Depressive symptoms were measured by using the Beck Depression Inventory II (BDI‐II), a 13‐item questionnaire. A total score of 10–19 indicates a mild depression; 20–29 a moderate depression; a score >30 indicates a severe depressive state (Beck et al., 1961). The Hamilton Rating Scale for Anxiety (HAM‐A) was used to measure the severity of anxious symptoms. It is a 14‐item scale, which measures psychic and somatic anxiety. The score ranges from 0 (not present) to 4 (severe): with a total score range of 0–56, where and <17 indicates mild anxiety, 18–24 moderate anxiety, and 25–30 severe anxiety (Beck et al., 1988). The Clinical Insight Rating Scale (CIRS) was used to evaluate levels of awareness of cognitive, functional impairments, disease progression, and reason for the visit. The overall score varies from 0 to 8, and each item can have a score from 0 to 2 (Ott & Fogel, 1992). We used the Body Image Scale (BIS), a 10 item questionnaire, to investigate different dimensions of body image in ABI patients. Each question is rated on 3 point Likert scale (0 = not at all; 3 = very much). The final score is the sum of the 10 items, ranging from 0 to 30, with zero score representing absence of symptoms or distress, and highest scores represent increasing symptoms and distress or more body image concerns (Cheli et al., 2016).

The Human Figure Drawing (HFD) was used to measure personality (Machover, 1951). All patients were asked to draw a human figure. After the first drawing, they were asked to draw another human figure that was of the opposite sex, to obtain a male HFD and a female HFD. We analyzed 43 details related to the human figure (including physical characteristics, drawing line, etc.). Each detail is rated as 0–1, where 1 indicates the presence of the detail whereas 0 the absence. In this way, it was possible to have a total HFD score of the male and female drawings. High scores correspond to adequate implicit body representation.

### 2.1 Statistical analysis

The Shapiro normality test was carried out to analyze the distribution of the variables. Continuous variables were expressed as Mean ± standard deviation, whereas in median, and first‐third quartile in no normal distribution. The Student's unpaired *t* test and the Mann–Whitney *U* test were used to compare two group (traumatic and vascular groups), when appropriate. For intragroup analysis, correlations between variables were computed by Pearson correlation or Spearman's coefficient. We performed a multiple regression analysis on the test HFD male, HFD female, and HFD total (dependent variables). At first, we focused on the influence of demographic and clinical variables, by using patient's age, education, and subitems of MoCA as predictors. We applied a backward elimination stepwise procedure for the choice of the best predictive variables according to the Akaike information criterion (AIC). Analyses were performed using an open source R3.0 software package. A 95% of confidence level was set with a 5% alpha error. Statistical significance was set at *p* < .05.

## 3 RESULTS

**FIGURE 1:**
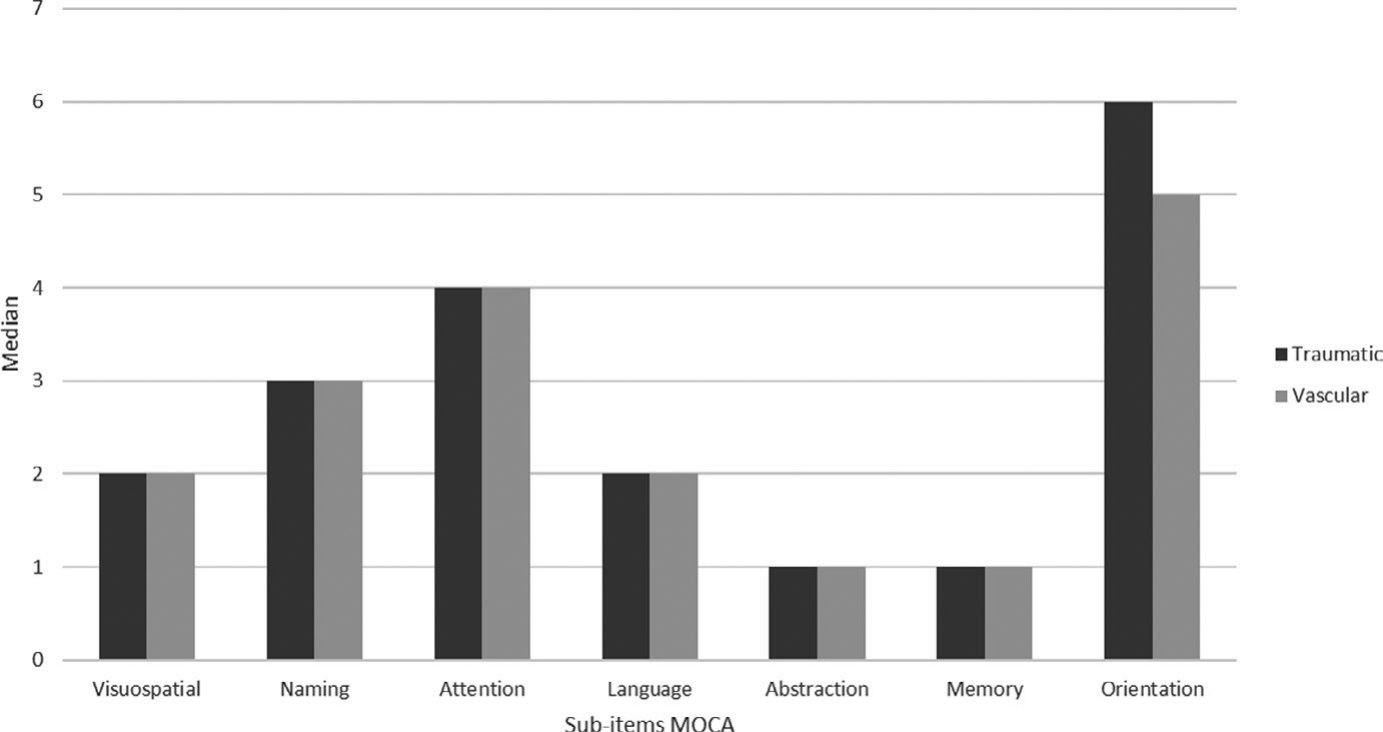
Representation by the histogram of subitems MoCA in traumatic and vascular groups

Descriptive analysis showed no significant differences between traumatic and vascular groups in both demographic and clinical scores (*p* > .05) (Table 1; Figure 1).

**TABLE 1:** Socio‐demographic characteristics of the traumatic and vascular groups

	Traumatic	Vascular	*p*‐value
Participants	17 (36.96)	29 (63.04)	–
Age	54.76 ± 22.03	55.66 ± 15.53	.88
Education	4.0 (3.0–4.0)	3.0 (3.0–4.0)	.32
Males	9 (52.94)	17 (58.62)	.94
Location of the lesion			.63
Right	7 (41.18)	15 (51.72)	
Left	7 (41.18)	8 (27.59)	
Bilateral	3 (17.64)	6 (20.69)	
MoCA	18.65 ± 5.43	17.90 ± 6.22	.67
BIS	6.0 (1.0–17.0)	7.0 (2.0–12.0)	.89
HAM‐A	9.0 (6.0–22.0)	12.0 (7.0–15.0)	.70
BDI‐II	13.29 ± 7.43	15.55 ± 8.87	.36
CIRS	2.0 (1.0–3.0)	2.0 (1.0–3.0)	.90
HFD male	18.0 (8.0–21.0)	18.0 (13.0–24.0)	.34
HFD female	16.0 (6.0–21.0)	17.0 (12.0–23.0)	.68
HFD total	32.0 (13.0–42.0)	34.0 (25.0–46.0)	.52

Note

Continuous variables were expressed as mean ± standard deviation when presented normal distribution, as median (first‐third quartile) otherwise. Categorical variables were expressed as frequency (percentage).

Abbreviations: BDI‐II, Beck Depression Inventory II; BIS, Body Image Scale; CIRS, Clinical Insight Rating Scale; HAM‐A, Hamilton Rating Scale for Anxiety; HFD, Human Figure Drawing; MoCA, Montreal Cognitive Assessment.

**FIGURE 2:**
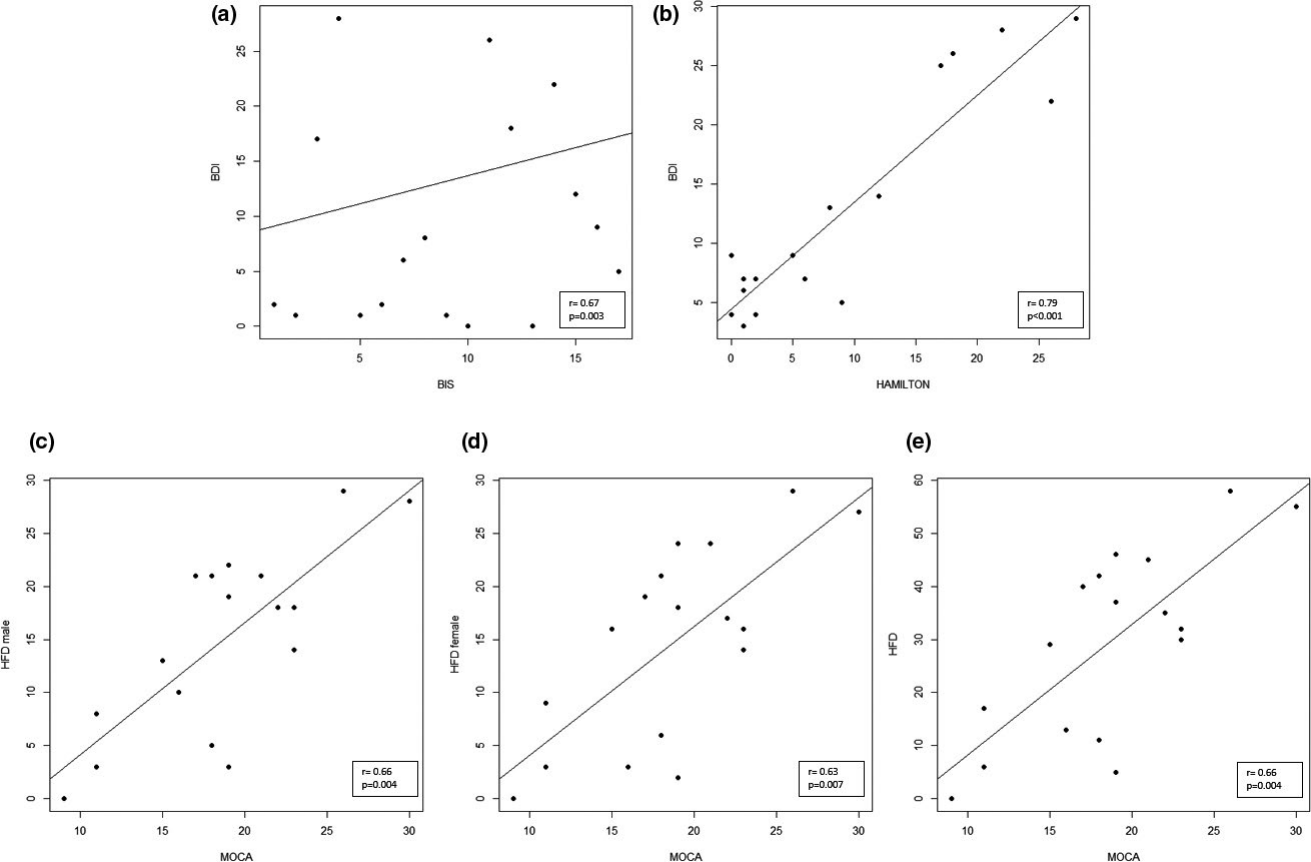
Correlation in the traumatic group. (a) Scatter plot of BIS score and BDI‐II score. (b) Scatter plot of BIS score and HAM‐A score. (c) Scatter plot of MoCA score and HFD male score. (d) Scatter plot of MoCA score and HFD female score. (e) Scatter plot of MoCA score and HFD score. BDI‐II, Beck Depression Inventory II; BDI‐II, Beck Depression Inventory II; HFD, Human Figure Drawing

In traumatic group, we found a significant positive correlation between BIS and BDI‐II score (*r* = .67; *p* = .003), as well as between BIS score and HAM‐A score (*r* = .79; *p* < .001). In addition, we observed some positive correlations: between MoCA and HFD male (*r* = .66; *p* = .004), MoCA and HFD female (*r* = .63; *p* = .007), and MoCA and HFD total (*r* = .66; *p* = .004) (Figure 2). The BIS scores correlate with the memory scores of MoCA (*r* = −.62; *p* = .008). We also found a trend between BIS and language (*r* = .47; *p* = .06) and abstraction (*r* = −.62; *p* = .008) MoCA subitems.

**FIGURE 3:**
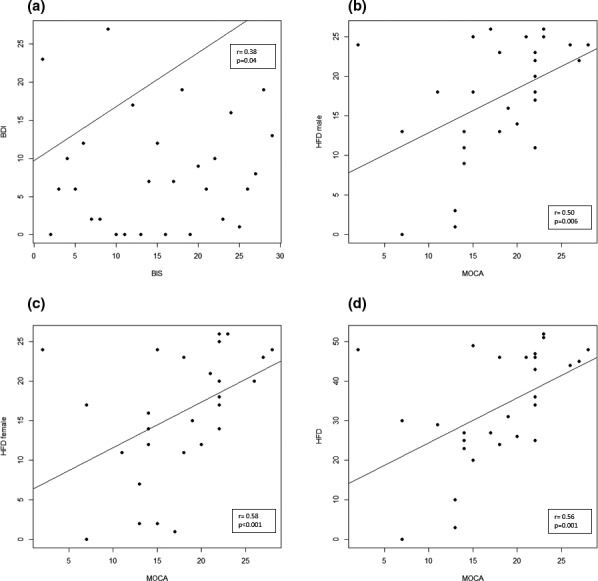
Correlation in the vascular group. (a) Scatter plot of BIS score and BDI‐II score. (b) Scatter plot of MoCA score and HFD male score. (c) Scatter plot of MoCA score and HFD female score. (d) Scatter plot of MoCA score and HFD score. BDI‐II, Beck Depression Inventory II; BDI‐II, Beck Depression Inventory II; HFD, Human Figure Drawing

In vascular group, we found a significant positive correlation between BIS and BDI‐II (*r* = .38; *p* = .04), as between MoCA and HFD male (*r* = .50; *p* = .006), MoCA and HFD female (*r* = .58; *p* < .001) and MoCA and HFD total (*r* = .56; *p* = .001). Results also highlighted a trend between BDI‐II and HFD female (*r* = −.33; *p* = .07) (Figure 3). No significant correlation between BIS and MoCA or its subitems emerged.

Multiple regression analysis showed that age has a significant impact on scores of HFD male in traumatic group. Visuospatial score was a significant predictor for HFD (male, female, total) in both traumatic and vascular groups (Tables 2 and 3).

**TABLE 2:** Backward linear regression: predictors on test HFD in the traumatic group

Dependent variables	Predictors	*β*	Std *β*	*p*‐value	Adjusted *R* ^2^
HFD male	Visuospatial	4.31	0.83	.009	0.75
	Age	−0.17	−0.43	.09	
	Education	−4.02	−0.6	.007	
HFD female	Visuospatial	4.33	0.79	.03	0.55
	Education	−3.54	−0.51	.05	
HFD total	Visuospatial	8.64	0.82	.02	0.66
	Education	−7.56	−0.56	.02	

Abbreviations: HFD, Human Figure Drawing; Std *β*, standardized regression coefficient; *β*, regression coefficient.

**TABLE 3:** Backward linear regression: predictors on test HFD in the vascular group

Dependent variables	Predictors	*β*	Std *β*	*p*‐value	Adjusted *R* ^2^
HFD male	Visuospatial	2.27	0.43	.04	0.48
	Naming	−3.18	−0.34	.06	
	Language	4.81	0.48	.02	
	Abstraction	−5.07	−0.52	.02	
	Orientation	1.81	0.35	.06	
HFD female	Visuospatial	2.87	0.51	.03	0.38
HFD total	Visuospatial	5.15	0.52	.01	0.50

Abbreviations: HFD, Human Figure Drawing; Std *β*, standardized regression coefficient; *β*, regression coefficient.

In addition in vascular group naming, language, abstraction, and orientation were significant predictors for HFD male.

## 4 DISCUSSION

The purpose of the present study was to evaluate the existence of correlations between body image and levels of anxiety, depression, and cognitive impairments in ABI patients; assessing differences in outcomes by brain damage etiology. Our findings show a positive correlation of BIS with BDI‐II and HAM‐A in traumatic group, as well as a positive correlation between BIS and BDI‐II in vascular group. This difference could be explained by a difference into vascular group due to the greater presence of depressive symptoms compared to anxious symptoms (Ayerbe et al., 2013; Campbell Burton et al., 2013). Schöttke and Giabbiconi (2015) showed a prevalence of 31.1% for depressive symptoms and a prevalence of 20.4% for anxious symptoms. The vascular group could be significantly more depressed than anxious, compared to the traumatic group. After all, Secord and Jourard (1953) showed that body dissatisfaction is associated with high levels of anxiety. Body image correlates significantly with affective disorders (Anderson & Antonak, 1992). Literature studies reported the presence of depressive symptoms associated with stroke (House, 1996). This emotional reaction following a vascular event may occur during acute phase of the disease or during hospitalization and rehabilitation (Langer, 1994).

Zhang et al. (2016) compared cognitive decline between TBI and stroke patients about executive performance, visual‐spatial, orientation, and memory. Their results showed that the TBI group showed significantly lower scores in orientation and memory than vascular group. Our findings are in line with this study, showing a relation between BIS and some MoCA domain as memory, language, and abstraction, but only in the traumatic group. In fact, stroke and TBI patients showed different cognitive impairment in mechanisms and clinical manifestations (Arciniegas et al., 2002).

Many studies showed that body representation can be altered because of brain injuries (Schwoebel & Coslett, 2005). The study conducted by Llorens et al. (2017) showed that stroke leads to a disorder of the body pattern, due to motor deficits reported by these patients. Razmus (2017) considered the relationship between body representation and general neuropsychological functions. The study confirmed that participants with cerebral vascular damage have deficits in body representation associated with neuropsychological deficits (working memory). In addition, a relationship between visual‐spatial skills and body image was highlighted in the localization tasks of body parts. Maresca et al. (2019) showed that both males and females with ABI, who are dissatisfied with their body image, achieve significant positive changes in the perception of many parts of the body associated with an improvement of cognitive and emotional deficits. Our study confirms the relationship between body image and cognitive deficits in ABI patients. We showed a positive correlation between MoCA and both HFD male and female, as well as with HFD total, in both traumatic and vascular groups. In particular, we found that some clinical conditions have a significant impact on scores of HFD (male, female, total). Indeed, we found that visuospatial score was a significant predictor on HFD (male, female, total) in traumatic and vascular group. In the vascular group, we also found some cognitive abilities (naming, language, abstraction, orientation) as predictive variables on HFD. We suppose that these findings could be due to a difference in the type of damage. Indeed, whether we hypothesize that in the traumatic group the damage reported was probably focused in specific cerebral areas, while in the vascular group, it could be a widespread damage involving more cerebral areas and therefore more cognitive domains. Regardless of this differentiation, our results confirm the crucial role of the cognitive level and emotional state on self‐perception.

The main limitation of our study is the small sample size and that all patients were hospitalized. Following these considerations, we believe that future research should be conducted with the enrollment of more subjects, who will be evaluated after hospitalization about the status of their cognitive functions, as well as bodily self‐perception.

We propose that neuropsychological rehabilitation can be useful for these patients not only to improve their cognitive performance, but also to have a more awareness of themselves. Psychotherapy is useful for patients to manage emotional dysfunction and increase a better body image perception, improving their quality of life.

## CONFLICT OF INTEREST

The authors declare that they have no conflicts of interest, including financial, consultant, institutional, and other relationships.

### PEER REVIEW

The peer review history for this article is available at https://publons.com/publon/10.1002/brb3.2181.

## Data Availability

Data are not available for this study given that account holders could be identifiable if it were made accessible.
